# Determinants of term premature rupture of membrane: case-control study in Saint Paul’s Millennium Medical College Hospital, Addis Ababa, Ethiopia

**DOI:** 10.1186/s12905-023-02497-8

**Published:** 2023-07-25

**Authors:** Zelele daniel, Temesgen Tantu, Dereje Zewdu, Thomas Mekuria, Tsion Yehualashet, Muluken Gunta, Mekete Wondosen

**Affiliations:** 1 Department of Obstetrics and Gynecology, College of Medicine and Health Science, Wolkite University, Wolkite, Ethiopia; 2 Department of Anesthesia, College of Medicine and Health Science, Wolkite University, Wolkite, Ethiopia; 3 Department of Obstetrics and Gynecology, Saint Paul’s Millennium Medical College, Addis Abeba, Ethiopia; 4 Department of Internal Medicine, College of Medicine and Health Science, Wolkite University, Wolkite, Ethiopia; 5 HIV officer at Wolayta Zone Health Department, Wolayta Sodo, Ethiopia; 6 Department of Surgery, College of Medicine and Health Science, Wolkite University, Wolkite, Ethiopia

**Keywords:** PROM, Puerperal sepsis, Clinical amnionitis, ANC, Short interpregnancy

## Abstract

**Background:**

The term premature rupture of the membranes is the rupture of the membranes before the onset of labor beyond 37 weeks of gestation. Several factors, including obstetric, gynecologic, socioeconomic, and medical, are identified as potential risk factors. This clinical event has detrimental maternal and neonatal complications.

**Objectives:**

This study aimed to investigate the determinants of the term premature rupture of the membranes in Ethiopia.

**Methods:**

This institution-based unmatched case-control study was conducted on 246 women admitted to Saint Paul’s hospital millennium medical college from October 2019 to January 2020 (82 cases and 164 controls). Data were collected using an interviewer-based questionnaire and data extraction tools, and data were entered using Epi data 3.1 and analyzed using SPSS 20. The association between independent variables and premature rupture of the membrane was estimated using an odds ratio with 95% confidence intervals and P-value < 0.05.

**Results:**

Factors like a history of vaginal discharge (AOR 3.508;95% CI:1.595.7.716), place of Antenatal care follow-up (health center and Mercy Ethiopia) (AOR 5.174;95% CI:2.165,12.362), the previous history of rupture of membrane (AOR 9.955;95% CI:3.265,20.35), and gestational age (AOR 3.018;95% CI:1.338,6.811) were associated with term premature rupture of membrane. There were more maternal and neonatal complications, including puerperal sepsis, wound infection, anemia/PPH, a hospital stays of more than seven days, clinical amnionitis, neonatal hypoglycemia, early onset neonatal sepsis, and respiratory distress encountered by women who presented with premature rupture of membrane.

**Conclusion:**

Proper screening, close monitoring, and early interventions in those mothers with identified risk factors would help to reduce its negative consequences. Moreover, the provision of continuous professional skill development and improving the quality of ANC service is needed.

## Background

Premature rupture of membrane (PROM) is the medical term for when amniotic membranes break before labor has begun. The fetal membrane comprises the chorion, the inner and outer amnion: the amnion gives the fetal membranes almost total tensile strength. As a result, the growth of the components that prevent rupture or ripping is essential for a successful pregnancy.

Physiologic rupture of the membrane occurs during or an hour before labor [1–4].

There have been sub-classifications for PROM based on gestational age. When the gestational age at the time of membrane rupture is greater than 37 weeks, it is term PROM. In the same way, considering preterm PROM is crucial when the incident occurs before 37 weeks of gestation. Gestational age is utilized as a cut-off point for sub-classification because it is the only variable affecting the outcome of the newborn.

Globally and particularly in the US, prematurity remains the leading cause of neonatal morbidity and mortality [5]. The occurrence of water breaks before 28 weeks of pregnancy is not recognized as PROM: because it is considered abortion under Ethiopian law. Patient history, clinical findings, and laboratory testing are helpful for the diagnosis of PROM [1, 2, 6, and 7].

Although the prevalence of PROM varies globally [8–12], it is estimated to complicate 3–10% of pregnancies, with the term PROM accounting for about a third of these complications [1–3]. Many studies reported that the prevalence of PROM in Ethiopia ranges from 1.4 to 25.8% [13–18]. The lack of adequate explanations of the pathophysiology and mechanisms behind PROM in the literature makes the quest for an effective cure difficult [1, 2]. However, several factors, including a history of abortion and PROM, smoking, vaginal discharge, inter-partum intervals, parity, abnormal vaginal discharge, polyhydramnios, urogenital infections, and the presence of ANC, are connected to the occurrence of membrane rupture [6–17].

Prematurity is the leading cause of neonatal mortality and morbidity worldwide. PROM accounts for more than 40% of preterm births, 18–20% of perinatal mortality, and 21.4% of perinatal morbidity. It considerably impacts perinatal mortality and morbidity in high- and low-income countries. Furthermore, various studies reported its association with maternal complications like intra amniotic infection, puerperal sepsis, placental abruption, a higher risk of developing disseminated intravascular coagulopathy and operative delivery, a rarely occurring retained placenta, and maternal death from sepsis [8, 11, 19, 20].

Similarly, the burden of PROM is high in our country; however, there are still no reliable methods for prediction and prevention. Identifying the determinants of PROM is vital to further elaborating and understanding it. To our knowledge, no study examined the determinants of PROM in the catchment area of Saint Paul’s Millennium Medical College.

Therefore, we sought to identify the determinants of PROM among mothers admitted to Saint Paul’s hospital millennium medical college, Ethiopia.

## Methods

### Study settings, design, and period

An institution-based, unmatched case-control study was conducted on women who gave birth at Saint Paul’s Hospital Millennium Medical College (SPHMMC) from October 2019–January 2020. The SPHMMC, located in Ethiopia’s capital city of Addis Ababa, has an inpatient capacity of more than 700 beds and an average of 1200 emergency and outpatient clients daily. The hospital provides delivery services 24 h per day and 7 days per week with midwives, interns, residents, obstetricians, and gynecologists on duty. The average number of deliveries per month is 800–900. All mothers admitted to the SPHMMC labor ward during study time and who fulfilled the inclusion criteria were the study populations.

### Sample size and sampling procedures

All mothers admitted to SPHMMC for labor/delivery was the study population. Mothers diagnosed with a ruptured membrane at term gestation and received management were cases, and mothers admitted to the labor ward without rupture of membranes were the control. The sample size was determined using the double population proportion formula for the case-control study design using Epi Info version 3.1 statistical software with consideration of the following assumptions: The power of the study was 80%, the confidence interval was 95%, the case-to-control ratio was 1 to 2, and the proportion of women with a history of abortion was 31.2% in patients with PROM and 10.6% in controls from one study done in Mekelle public hospitals [1]. The calculated sample size was 246 (82 cases and 164 controls). All pregnant women with and without PROM at 37 weeks of gestational age were included in the study.

The recruitment of women with PROM who met the criteria for inclusion continued until we reached the calculated sample size for cases.

Those mothers without rupture of membranes admitted before and following the cases were selected using a simple random sampling technique as controls.

#### Inclusion criteria

##### Cases

All term pregnant mothers admitted to the labor ward with ruptured membranes.

##### Controls

all mothers admitted to the labor ward with normal labor.

#### Exclusion criteria

All pregnant mothers admitted for diagnosis of obstetric and medical complications like multiple pregnancies, congenital anomalies, intrauterine fetal death, intrauterine growth restriction, post-term pregnancy (admitted electively), pre-eclampsia, eclampsia, diabetes, or chronic hypertension.

#### Definitions

##### Short interpregnancy interval

Index pregnancy occurred less than 18 months after the preceding pregnancy [2, 3].

##### Optimal inter-pregnancy intervals

Index pregnancy occurring within 18–59 months of preceding pregnancy [2, 3].

##### Longer interpregnancy intervals

Index pregnancy occurring beyond 60 months of preceding pregnancy [2, 3].

### Data collection tools, methods, and personnel

The mothers were informed about the purpose and usefulness of the study.

We received informed written consent from the mothers before data collection. Two trained midwives, who had previous experience in data collection, collected the data, and the principal investigator was involved in supervision. Data were collected using interviews and relevant data from medical records. For the first seven days, neonates admitted to the NICU and staying in the maternity ward for maternal indications were monitored for the possible development of the other outcome variables throughout the course. Data collectors (midwives) were assigned to follow neonates discharged early until the seventh day of life, with a phone interview with the mother about the neonate: whether the neonate was breastfeeding, sleeping well, alert, and if there was any problem, the mother had to bring it back. After reviewing various works of literature, we developed a well-structured questionnaire checklist in English. The questionnaire checklist contains variables including; socio-demographics, obstetric history, and medical and maternal-fetal outcomes.

We performed a pre-test using 5% of the sample size (4 cases and 8 controls) at Zewditu Hospital in Addis Ababa. The questionnaire’s internal consistency was checked using Cronbach’s alpha of 0.79. After making some corrections based on the pre-test results, the data collection commenced. The principal investigators and supervisors supervised the data collection process daily for completeness.

#### Standard and operational definitions

Early ultrasound: the ultrasound taken before the gestational age of 24 weeks (12, 13).

Abortion: when the pregnancy is terminated before the gestational age of 28 weeks. (15)

### Data analysis

Data were extracted, coded, and entered into the Epidata 3.1 program for cleaning before being exported to SPSS 20 for additional analysis and report writing. Simple frequencies, crosstabs, means, and standard deviation were used in the descriptive statistical analyses to summarize participant sociodemographic data, pregnancy-related variables, and labor and delivery-related features. The association between PROM and the independent factors was investigated using binary logistic regression with 95% CI. To further enhance the analysis, variables with p values less than 0.25 in bivariate analysis were included in multivariate analysis. We computed multivariable logistic regression analysis to assess the associations between the PROM and significantly associated independent predictors. P-value < 0.05 was considered statistically significant. Model fitness was measured using the Hosmer and Lemeshow goodness of fit measures and the Nagelkerke R square, which were 0.64 and 0.58, respectively. The variance inflation factor (VIF > 10) was used to test for multicollinearity between the explanatory variables.

## Results

### Socio-demographic characteristics of study participants

A total of 246 study participants (82 were cases and 164 were controls) were involved making the response rate 100%. The mean age of cases and controls was 25.96 (± 4.8) years and 26.179(± 4.3) years, respectively. The majority of cases (89%) and controls (92.7%) were between the age of 19–35. Regarding marital status, almost all women in the case group (91.5%) and control group (91.5) were married as well as only one in case but no in controls: single. Nearly half of the women in cases (45.1%) and control (47.6%) were housewives. (Table 1)

**Table 1:** Socio-demographic characteristics of study participants

Variable		Frequency		X^2^^(P−value)^
		Cases	Controls	
Age Category	< 19	2(2.4%)	3(1.8%)	0.784(0.676)
	19–35	73(89%)	152(92.7%)	
	> 35	7(8.6%)	9(5.5%)	
Marital Status	Married	75(91.50%)	150(91.50%)	2.1(0.350)
	Unknown*	6(7.30%)	14(8.5%)	
Education level	No formal education	7(8.5%)	18(11.0%)	1.115(0.892)
	Primary school	33(40.2%)	71(43.3%)	
	Secondary school	23(28.1%)	37(22.6%)	
	Above secondary school	12(14.6%)	24(14.6%)	
	Unknown*	7(8.5)	14(8.5%)	
Occupation	Housewife	37(45.1%)	78(47.6%)	3.129(0.680)
	Merchant	11(13.4%)	16(9.8%)	
	Government employee	22(26.8%)	34(20.7%)	
	Others**	3(3.7%)	9(5.5%)	
	Unknown*	5(6.1%)	15(9.1%)	
	Daily laborer	4(4.9%)	12(7.3%)	
Residence	Addis Ababa	51(62.2%)	100(61.0%)	0.034(0.853)
	Outside of Addis Ababa	31(37.8%)	64(39.0%)	

* = if there is no information about it. **= other than mentioned (private or self-employment)

### Obstetric and health-related characteristics of respondents

A slight variation was noticed regarding gravidity: the controls (39.5%) and half of the cases (50%) were primigravidas. Based on parity, more than half of the cases (58.5%) and controls (51.2%) have one delivery. Furthermore, the inter pregnancy interval is less than 18 months in 24.4% of cases and 2.4% of controls. Around half of the cases (48.8%) and 29.3% of controls have at least one episode of abortion; spontaneous abortion comprises the majority of abortions, with 40 (83.3%) in controls and 29 (72.5%) in cases. One-third of cases (37.8%) had a previous history of rupture of the membranes, but only 9 (5.5% of controls) had it. All participants had ANC follow-ups, but the location of the follow-ups differed. (Table 2)

**Table 2:** Obstetric and health service-related Characteristics of Respondents Admitted to Saint Paul’s hospital millennium medical college, Addis Ababa

Variables		controls (164)	Cases (82)	total	x2 (p-value)
Gravidity	One	65(39.6%)	41(50%)	106	2.84(0.241)
	Two –four	94(57.3%)	40(48.7%)	134	
	Greater than or equal to five	5((3.1%)	1(1.3%)	6	
Parity	Zero	84(51.2%)	48(58.5%)	132	2.51(0.281)
	One	42(25.6%)	22(26.8%)	64	
	2 to 4	38(23.1%)	12(14.7%)	50	
Pregnancy Type	Planned and wanted	98(59.8%)	54(65.9%)	152	0.861(0.353)
	Unplanned but wanted	66(40.2%)	28(34.1%)	94	
Interpregnancy interval in months	Less than 18 months	4(2.4%)	20(24.4%)	24	51.9(0.00)
	Optimal interval	5(3.1%)	14(17%)	19	
	Long interval	20(12.2%)	2(2.4%)	22	
	Not applicable	121(73.8%)	42(51.2%)	163	
	Unknown	14(8.5%)	4(4.9%)	18	
History of abortion	Yes	48(29.3%)	40(48.8%)	88	9.09(0.003)
	No	116(70.7%)	42(51.2%)	158	
History of PROM	Yes	9(5.5%)	31(37.8%)	40	42.9(0.00)
	No	142(86.6%)	44(53.7%)	186	
	Unknown	13(7.9%)	7(8.5%)	20	
Type of abortion experienced	Spontaneous	40(24.4%)	29(35.4%)	69	1.71(0.191)
	Induced	8(4.9%)	11(13.4%)	19	
ANC follow up	Yes	164(100%)	82(100%)	246	
Place of ANC follow up	SPHMMC	71(43.3%)	13(15.9%)	84	20.23(0.00)
	Health center	89(54.3%)	64(78%)	153	
	Mercy care Ethiopia	2(1.2%)	4(4.9%)	6	
	Private hospital or clinic	1(0.6%)	1(1.2%)	2	
The gestational age in weeks	37 to 38weeks + 6 days	23(14%)	29(35.4%)	52	14.18(0.001)
	39 to 42 weeks	115(70.1%)	45(54.9%)	160	
	> 42 weeks	22(13.4%)	8(9.8%)	30	

### Clinical and medical conditions of the respondents

Forty-three percent of the participants in the case group had a history of vaginal discharge, and only 13.4% of controls had it. There was a comparable number of participants with polyhydramnios, in both cases (2.4%) and control groups (1.8%). 12% of cases and 4.3% of control had a history of pregnancy-induced hypertension during the current pregnancy. (Table 3)

**Table 3:** Clinical and Medical Conditions of Respondents Admitted Saint Paul’s hospital millennium medical college, Addis Ababa

variables		controls (164)	Cases (82)	Total (244)	x2 (p-value)
History of abnormal vaginal discharge during this pregnancy	Yes	22(13.4%)	36(43.9%)	58	28.5(< 0.01)
	No	128(78%0.1)	40(48.8%)	168	
	Unknown	14(8.5%)	6(7.3%)	20	
Having a chronic cough	yes	5(7.8%)	2(2.4%)	7	3(0.81}
	no	159(92.2%)	80(97.6%)	239	
Hemoglobin(gm/dl)	≥ 11	7(4.3%)	9(11%)	16	6.15(0.46)
	11	157(95.7%)	73(89%)	230	
Diagnosed polyhydramnios	yes	3(1.8%)	2(2.4%)	5	1.1(0.99)
	no	163(98.2%)	80(97.6%)	243	
MUAC(Cm)	< 23	4(2.4%)	6(7.3%)	10	2.3(0.45)
	≥ 23	160(97.6%)	76(92.6%)	236	
Having Pregnancy-induced hypertension (PIH)	yes	7(4.3%)	10(12%)	17	4.65(0.33)
	no	157(95.7%)	72(88%)	229	

### Risky physical activities and habits among respondents

A few individuals reported a smoking history (7.3% in cases, 3% in control) and alcohol usage (8.5% in cases, 5.5% in control). Only one individual in the control group reported an accidental fall in index pregnancy but no report in the cases group. (Table 4)

**Table 4:** Risky Physical Activities and Habits among Respondents Admitted to Saint Paul’s hospital millennium medical college, Addis Ababa

variables		controls (164)	Cases (82)	Total (246)	x2 (p-value)
Smoking	yes	5(3%)	6(7.3%)	11	3.67(0.81}
	no	159(97%)	76(92.7%)	235	
Alcohol consumption	yes	9(5.5%)	7(8.5%)	16	6.15(0.46)
	no	155(94.5%)	75(91.5%)	230	
Heavy load lifting	yes	3(1.8%)	2(2.4%)	5	0.88(0.99)
	no	163(98.2%)	80(97.6%)	243	
Having help at home	yes	100(61%)	53(64.6%)	153	22(0.05)
	no	64(39%)	29(35.4%)	93	
Accidental fall during pregnancy	no	163(99.4%)	82(100%)	245	0.21(0.63)
Having sexual intercourse in 3rd trimester	yes	10(6.1%)	3(1.8%)	13	0.34(0.77)
	No	154 (93.9%)	79 (98.2%)	233	

### Maternal and neonatal outcomes of study participants

Among the study participants, the complications encountered by women who presented with PROM were puerperal sepsis, wound infection, and anemia/PPH. These complications happened in a lower percentage among controls. And 1 case stayed for more than 7 days but no one in the control group. Regarding neonatal complications, 8.5% of the cases and 1.8% of the controls had faced hypoglycemia, EONS, and respiratory distress. (Table 5)

**Table 5:** Maternal and neonatal outcomes of study participants Admitted to Saint Paul’s hospital millennium medical college, Addis Ababa

Maternal and neonatal complications		controls (164)	Cases (82)	Total (246)	x2 (p-value)
Puerperal sepsis	no	164 (100%)	80 (97.6%)	244	
Wound infections	yes	1 (0.6%)	2 (2.4%)	3	
	no	163 (99.4%)	80 (97.6%)	243	
Duration of hospital stay	Less than 3 days	140 (85.4%)	51 (62.2%)	191	
	3-7days	46 (14.6%)	30 (36.6%)	76 1	
Anemia/PPH	yes	2 (1.2%)	2 (2.4%)	4	
	no	162 (98.8%)	80 (97.6%)	242	
Clinical chorioamnionitis	yes	1 (0.6%)	1 (1.2%)	2	
	no	163 (99.4%)	81 (98.8%)	244	
Neonatal hypoglycemia	No	164 (100%)	81 (98.8%)	165	
Early onset neonatal sepsis	Yes	1 (1.2%)	5 (6.1%)	6	
	No	162 (98.8%)	77 (93.9%)	239	
Respiratory distress	Yes	1 (0.6%)	1 (1.2%)	2	
	No	163 (99.4%)	81 (98.8%)	244	
unknown	Yes	(0.6%) 1	(1.2% 1)	2	
	No	163 (99.4%)	81 (98.8%)	244	

### Determinants of premature rupture of membranes (PROM)

All variables passed through a bivariate logistic analysis. Those variables with a p-value less than 0.25 in bivariate analysis were further analyzed using multivariate logistic analysis to check for the association. The following factors had a significant association with rupture of membranes in the bivariate analysis: gestational age at rupture of membranes, history of abortion, previous history of rupture of membranes, and place of ANC follow-up. The significantly associated variables listed above were exported to multivariate analysis for further investigation of the association. On multivariate analysis, the following factors were significantly associated with membrane rupture: history of vaginal discharge (AOR: 3.508 (95% CI: 1.595, 9.955), previous history of rupture of membrane (AOR: 9.955), follow-up at other institutions (health center, Mercy Ethiopia, and private clinic) (AOR: 5.174 (2.165, 12.362)), and gestational age (AOR: 3.93 (95% CI: 1.676, 9.22). ( Table 6)

**Table 6:** Determinants of PROM Among Women Admitted to Saint Paul’s hospital millennium medical college, Addis Ababa

variables		PROM		COR (95% CI)	AOR (95% CI)	p-value
		controls (164)	Cases (82)			
The gestational age in weeks	37 to 38wks + 6 days	23	29	3.22(1.688,6.1552)	3.018(1. 338,6.811)	0.008
	39 to 42 weeks	115	45	1	1	
	> 42 weeks	22	8	0.929(0.386,2.29)	0.401(0.122,1.314)	0.12
History of PROM	Yes	9	31	11.116(4.918,25.127)	9.955(3.265,20.35)	0.001
	No	142	44	1	1	
History of abortion	Yes	48	40	2.302(1.33,3.983)	0.903(0.375,2.173)	0.44
	No	116	42	1		
History of abnormal vaginal discharge during this pregnancy	Yes	22	36	5.236(2.766,9.913)	3.508(1.595,7.716)	0.002
	No	128	40	1	1	
Place of ANC follow up	SPHMMC	71	13	1	1	
	Other institutions**	91	69	4.09(2.099,7.994)	5.174(2.165,12.362)	0.001

**= health center, mercy care Ethiopia, and private clinics

## Discussion

This study found that pregnant women with a history of PROM have a 9.955 times greater likelihood of developing PROM than those whose mothers did not. In agreement with our findings, other studies conducted in Ethiopia, America, and Nigeria found the impact of a history of PROM on subsequent pregnancy [4–9].

The cause could be linked to pre-existing issues like a short cervix and an untreated genito urinary tract infection during the previous pregnancy and persisted into this one. Given the high likelihood of recurrence of obstetric difficulties, screening and ongoing follow-up would help women with a history of such complications.

Vaginal discharge syndrome is one of the common manifestations of genital tract infections, including chlamydia, Neisseria gonorrhea, bacterial vaginosis, and group B streptococcus. These infections are strongly associated with PROM in different Ethiopian, Nigerian, and Indian studies [1, 4, 9–11].

Hence, this study confirmed the above finding by showing the history of vaginal discharge as one of the determinant factors for PROM in the index pregnancy. Pregnant mothers with a history of vaginal discharge have odds of 3.508 times higher than mothers with no history of vaginal discharge. The above etiologic agents ascend to the cervix and tend to release inflammatory mediators, which can weaken the strength of the amniotic membranes during pregnancy, causing PROM. Additionally, intrauterine infections are secondary to ascending genital colonization with microbes, resulting in the release of cytokines, apoptosis, and finally, the dissolution of the collagens in amniotic membranes [12, 13].

Additionally, gestational ages between 37 + 0 and 38 + 6 weeks are strongly associated with PROM. Pregnant ladies with a gestational age of 37 + 1 to 38 + 6 weeks have almost three times higher odds of PROM than those with a gestational age of 39 + 0 to 40 + 6 weeks. This finding is in line with other results [4, 9, 21]. It has been demonstrated that the likelihood of PROM decreases with increasing gestational age [12, 14].

Fortunately, all pregnant women had ANC follow-up in this study, but the place of care is associated with PROM. Mothers who had their follow-up at a health center, private clinic, or Mercy Care Ethiopia had a 5.174 times higher risk of PROM than those who had their follow-up at SPHMMC. The health center is one of the lowest units in the health hierarchy in Ethiopia [15]. It has low-level health professionals who may not have the expertise to identify risk factors and/or have insufficient resources to assess the risk. Additionally, Mercy Care Ethiopia is a non-governmental institution giving maternal and child care services in Ethiopia and has the same problem as the health center in this issue.

Despite the lack of studies comparing the follow-ups between institutions, it is evident that pregnant women at high risk of PROM should receive follow-up and treatment in advanced centers in well-organized obstetric centers [12, 13].

There was a higher record of fetal and maternal problems among patients, which is concerning. The current study found that maternal complications included puerperal sepsis (2.4%), wound infection (2.4%), a hospital stay of > 7 days (1.2%), anemia/PPH (2.4%), and clinical amnionitis (1.2%), while fetal complications included neonatal hypoglycemia (1%), early onset neonatal sepsis (6.5%), and respiratory distress (1.2%). (1.2%). The result is consistent with other investigations [8, 18, 22].

### Limitations of the study

The study may be susceptible to recollection bias because it may be difficult to recall past events. The typical methods used in our nation to diagnose PROM are historical and clinical rather than laboratory-based, which makes the study susceptible to selection bias. In our setting, there are no investigative methodologies to diagnose PROM.

## Conclusion

This research revealed variables including prior PROM history, previous vaginal discharge, and follow-up at low-level healthcare facilities (health center and mercy care Ethiopia).

To reduce the likelihood of PROM, pregnant women with the recognized risk factors should be closely monitored and given proper treatment during the ANC follow-up. Health practitioners at low-level health facilities should get ongoing professional development for identifying the risk factors and consequences of PROM (health centers and mercy care).

## Data Availability

All data is available upon request from the corresponding author.
